# A Three Dimensional Study of Upper Airway in Adult Skeletal Class II Patients with Different Vertical Growth Patterns

**DOI:** 10.1371/journal.pone.0095544

**Published:** 2014-04-22

**Authors:** Tianhu Wang, Zhenhua Yang, Fang Yang, Mingye Zhang, Jinlong Zhao, Jinwu Chen, Yongming Li

**Affiliations:** 1 State Key Laboratory of Military Stomatology, Department of Orthodontics, School of Stomatology, The Fourth Military Medical University, Xi’an, Shaanxi, People’s Republic of China; 2 State Key Laboratory of Military Stomatology, Department of Oral and Maxillofacial Surgery, School of Stomatology, The Fourth Military Medical University, Xi’an, Shaanxi, People’s Republic of China; 3 State Key Laboratory of Military Stomatology, Department of Radiology and Intervention Therapy, School of Stomatology, The Fourth Military Medical University, Xi’an, Shaanxi, People’s Republic of China; Research Center Borstel, Germany

## Abstract

**Objective:**

The study was performed to compare the 3D pharyngeal airway dimensions in adult skeletal Class II patients with different vertical growth patterns (low, normal, and high angle) and to investigate whether the upper airway dimensions of untreated skeletal Class II adults were affected by vertical skeletal variables.

**Methods:**

Cone-beam computed tomography (CBCT) records of 64 untreated adult skeletal Class II patients (34 male and 30 female) were collected to evaluate the pharyngeal airway dimensions. Subjects were divided into three subgroups according to the GoGn-SN angle (low angle, normal angle or high angle). All subgroups were matched for sex. ANOVA and SNK - q tests were used to identify differences within and among groups (p<0.05). Coefficient of product-moment correlation (Pearson correlation coefficient) was used to analyze the association between pharyngeal airway dimensions and vertical growth patterns.

**Results:**

The results showed that pharyngeal airway measurements were statistically significantly less (p<0.05) in high angle group as compared to normal angle or low angle group.

**Conclusions:**

Adult skeletal Class II subjects with vertical growth patterns have significantly narrower pharyngeal airways than those with normal or horizontal growth patterns, confirming an association between pharyngeal airway measurements and a vertical skeletal pattern.

## Introduction

Obstructive sleep apnea (OSA) is a common respiratory sleep disorder characterized by snoring and episodes of breathing cessation during sleep despite respiratory effort, which is rarely observed in young subjects. OSA involves an occlusion of the upper airway, which typically lies at the level of the oropharynx and less often at the nasopharynx or hypopharynx. A reduction in pharyngeal space is a clinical observation commonly reported in OSA patients; however, until now, the precise mechanism of upper airway occlusion remains under debate [1]. Previous studies of OSA from different samples have shown an association between craniofacial skeletal morphology and upper airway dimensions in OSA patients [2]. It has been reported that Class II malocclusions and vertical growth patterns are anatomic predisposing factors for the obstruction of the pharyngeal airways [3]–[7]. Furthermore, when compared to healthy patients with normal occlusions and growth patterns, or Class I malocclusions, healthy patients with Class II malocclusions and vertical growth patterns might have narrower airway passages.

Regardless of OSA etiology, identifying the area of airway obstruction has often proved challenging. During the past a few decades, various methods have been used to evaluate the airway, including nasopharyngoscopy [8], cephalometry [9], nasal airway resistance [10], as well as polysomnography [11]. Lateral and frontal radiographs have been used to assess the pharyngeal airway [7], [12]–[14]. However, few of these studies have evaluated the full length and width of the airway in three dimensions. Cone beam computed tomography (CBCT), with three-dimensional (3D) presentation of the airway and its surrounding structures, makes volumetric analysis and accurate visualization of the airway possible [15]. By using CBCT scans to analyze the complex airway anatomy, previous studies [16]–[18] have confirmed that volumetric measurements of airways by utilizing CBCT are accurate and with minimal error, thus offering an increased view of both untreated obstruction tendencies and potential changes in the airway by treatment modality.

The aims of our present study were (1) to compare the 3D pharyngeal airway dimensions in adult skeletal Class II patients with different vertical growth patterns (low, normal, and high angle) and (2) to investigate whether the upper airway dimensions of untreated skeletal Class II adults were affected by vertical skeletal variables.

## Materials and Methods

This study protocol was approved by the Ethics Review Committee at the College of Stomatology, Fourth Military Medical University. It conforms to relevant national and international guidelines. Written informed consent was obtained from each patient. The study included CBCT scans of 64 healthy Han Chinese adults (34 male and 30 female), aged 20 to 35 years (mean age 26.3 years), who visited the Department of Orthodontics, College of Stomatology, Fourth Military Medical University, for orthodontic treatment between December 2008 and April 2010. Those who had symptoms of upper respiratory infection, pharyngeal pathology such as adenoid hypertrophy and tonsillitis or a history of adenoidectomy or tonsillectomy were excluded.

The selection criteria for the skeletal Class II facial pattern was ANB>5° (mean 5.8°). The subjects were then assigned to three groups based on their SN mandibular plane (GoGn-SN) angle as described by previous literature [19].

Subgroup 1: Low angle group, GoGn-SN angle lower than 22° (20 subjects)

Subgroup 2: Normal angle group, GoGn-SN angle between 22° and 32° (24 subjects)

Subgroup 3: High angle group, GoGn-SN angle higher than 32° (20 subjects).

**Figure 1:**
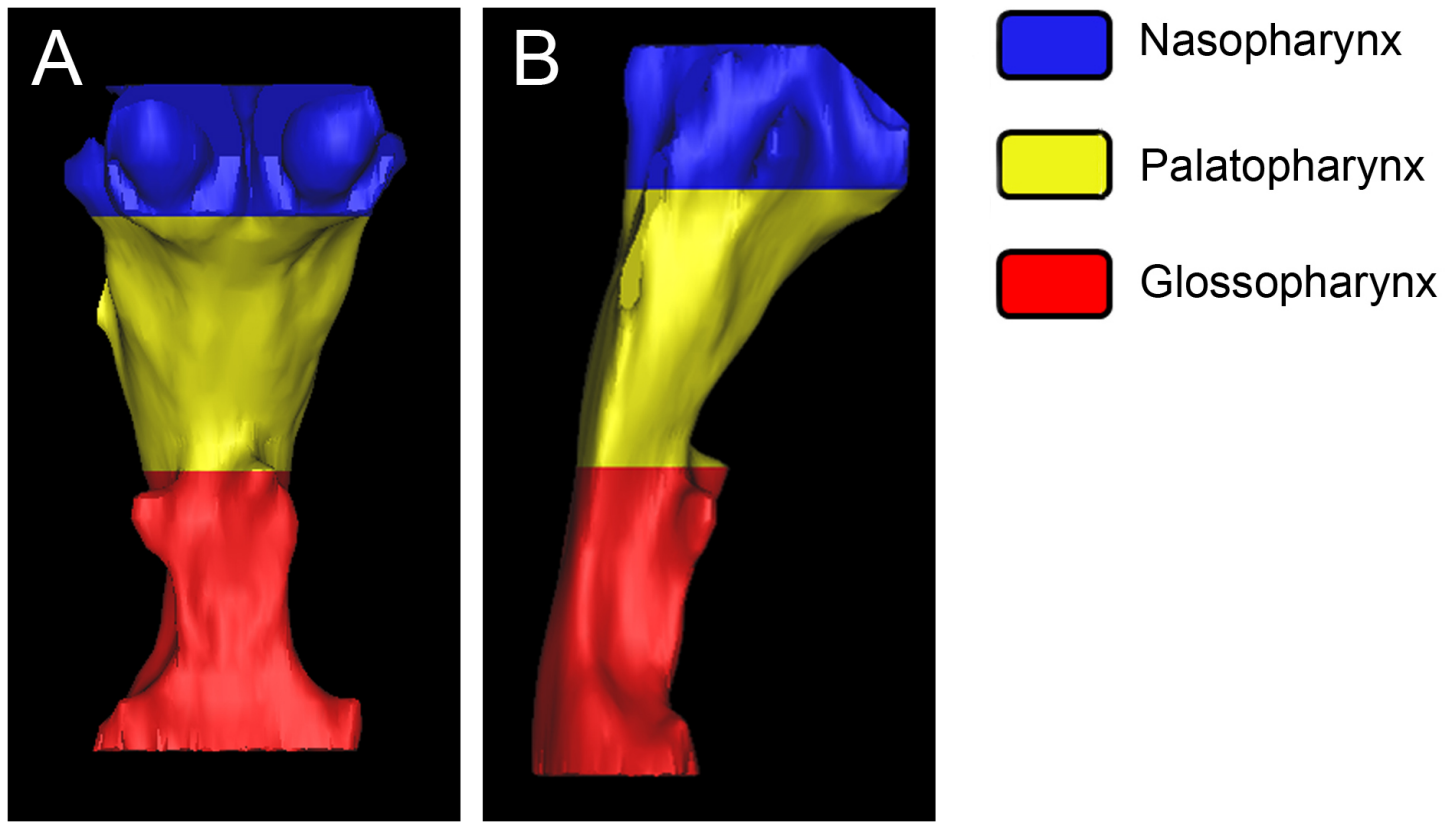
Three-dimensional upper airway model. A: frontal view; B: lateral view.

All the patients underwent a CBCT examination (GALILEOS, SIRONA, Bensheim, Germany) for orthodontic diagnosis and treatment planning. CBCT radiographs were exposed as patients stood upright with the Frankfort horizontal plane parallel to the floor. During radiography, subjects were asked to contact their teeth at maximum intercuspation to stabilize the mandible, hold their breath at the end of expiration and avoid swallowing to control the tongue. Images were taken at 21 mA, 85 kV, in a scanning field of 15 by 15 cm, and with 150 µm slice thickness for three dimensional (3D) reconstruction. All patients were scanned by the same radiology technicians in the Department of Radiology. Resulting CBCT images were stored in the workstation computer and converted into DICOM (Digital Imaging and Communication in Medicine) format. Each subject was scanned from the Frankfort horizontal line to the hyoid bone. Final optimized 3D pharyngeal airway models were acquired through Mimics 10.0 and autoCAD, and volumetric analysis of the defined airways was performed using Mimics 10.0 (Figure 1).

**Figure 2:**
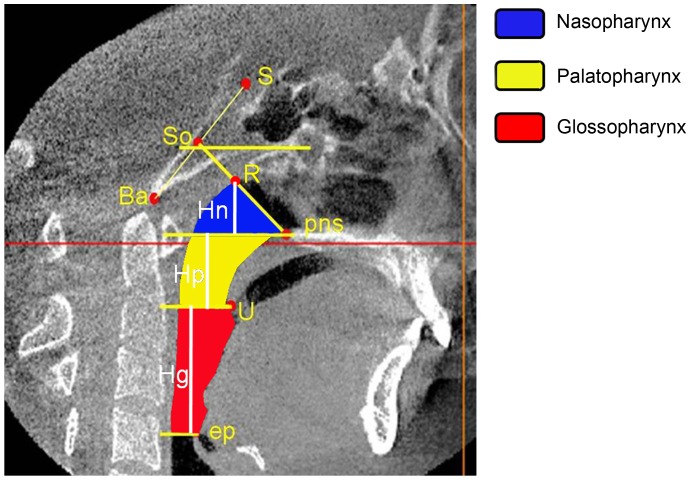
Reference planes and pharyngeal airway measurements used in this study.

**Figure 3:**
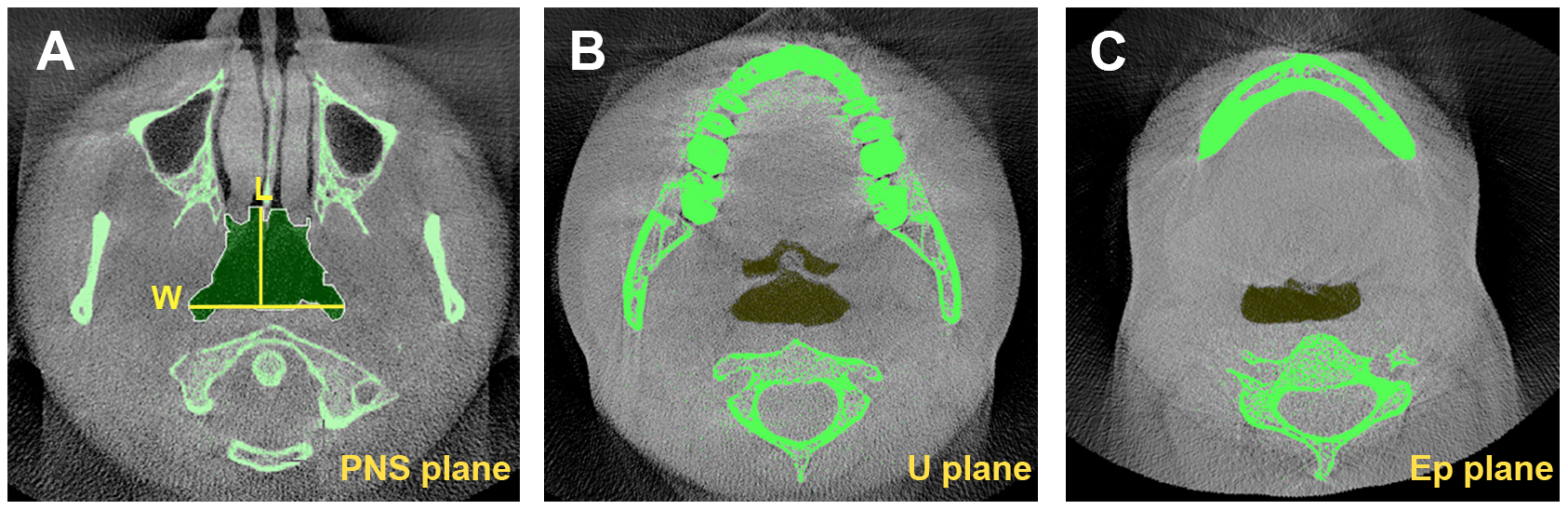
Cross-sectional views of the pharyngeal airway in the 3 planes. (A: PNS plane; B: U plane; C: Ep plane): L represents the linear sagittal length of the airway; W is the linear transverse width of the airway. The colored region indicates the cross-sectional area of the airway.

Landmarks and analysis were based on methods described previously [20], [21]. Landmarks and measurements identified in this study are outlined in Figure 2 and 3. Skull markers were as follows: S (sella); Ba (basion); So (midpoint of line segment S-Ba); PNS (posterior nasal spine); R (intersection point of line So-PNS and the superior border of nasopharyngeal airway); U (tip of the uvula); Ep (apex point of the epiglottis). Limits of the upper airway, including naso-, palato- and glossopharyngeal, were determined as follows: the anterior border of the nasopharyngeal airway (a plane passing through the PNS and R, perpendicular to the sagittal plane); the inferior border of the nasopharyngeal airway (also the superior border of the palatopharyngeal airway, a plane parallel to the FH plane through the PNS, perpendicular to the sagittal plane); the superior border of the glossopharyngeal airway (also the inferior border of the palatopharyngeal airway, a plane parallel to the FH plane through U, perpendicular to the sagittal plane); the inferior border of the glossopharyngeal airway (a plane parallel to the FH plane through Ep, perpendicular to the sagittal plane); the posterior border was the posterior wall of the pharynx.

**Figure 4:**
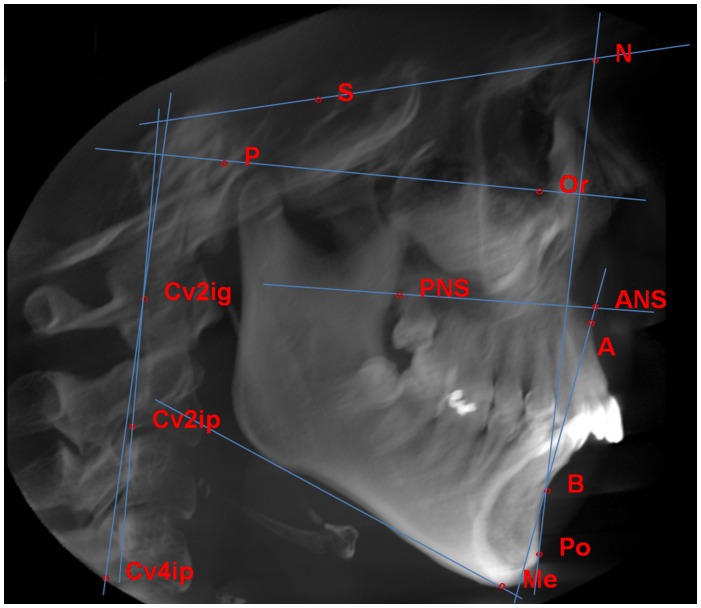
Landmarks involved in the vertical skeletal and head posture measurements on cephalograms from CBCT.

The vertical skeletal and head poster measurements identified on lateral cephalograms from CBCTs are outlined in Figure 4: Cv2ig (the most superior, posterior point on the second cervical vertebra); Cv2ip (the most inferior, posterior point on the second cervical vertebra); Cv4ip (the most inferior, posterior point on the fourth cervical vertebra); NSL, the line passing through N and S; OPT, the line passing through the most superior, posterior point on the second cervical vertebra and the most inferior, posterior point on the second cervical vertebra; CVT, the line passing through the most superior, posterior point on the second cervical vertebra and the most inferior, posterior point on the fourth cervical vertebra; OPT/NSL, the angle formed by OPT and NSL; CVT/NSL, the angle formed by CVT and NSL. Vertical skeletal measurements including FMA angle formed by Frankfort horizontal plane (FHP) and mandibular plane (Go-Me), SN mandibular plane (GoGn-SN) angle and the Overbite Depth Indicator (ODI) were as described previously [22], [23].

Volume and linear measurements used in current study: Vn,Vp,Vg and Vt represent nasopharyngeal (NA), palatopharyngeal (PA), glossopharyngeal (GL) and total upper airway volume, respectively (Figure 1); Hn, Hp and Hg represent naso-, palato- and glossopharyngeal height, respectively (Figure 2); CSAn, CSAp and CSAg represent the cross sectional area (CSA) measured at the segmented plane of the inferior border of the naso-, palato- and glossopharyngeal airway, respectively (Figure 3); Ln, Lp, Lg and Wn, Wp, Wg represent linear sagittal lengths and transverse widths measured at the segmented plane of the inferior border of the naso-, palato- and glossopharyngeal airway, respectively (Figure 3).

CBCT images of 15 randomly selected patients were measured again by the same investigator 10 days after the first measurements. Wilcoxon signed rank test was applied and the difference between the two measurements was not statistically significant (p>0.05).

Statistical procedures were performed on the recorded data using SPSS 17.0 software. One-way analysis of variance (ANOVA) was performed among the subgroups. When ANOVA was significant, further comprehensive comparison was done with SNK - q test and the a priori level of significance was set at P≤0.05. The association between upper airway dimensions and vertical growth patterns were analyzed by coefficient of product-moment correlation (Pearson correlation coefficient).

To control distribution of sex in balance, the following analyses were performed for the three subgroups. A chi-square test was performed to examine the sex distribution in each subgroup. No significant difference was detected (Table 1). Head posture was compared among the three subgroups (Table 2) with ANOVA, and no statistical difference was observed.

**Table 1:** Sex Distribution in the subgroups of subjects with different vertical skeletal pattern.

	L	N	H	Total	P
Male	11	13	10	34	>0.1
Female	9	11	10	30	
Total	20	24	20	64	

L indicates low angle group, N indicates normal angle group, H indicates high angle group.

**Table 2:** Head posture in the subgroups of subjects with different vertical skeletal pattern.

	Low Angle Group		Normal Angle Group		High Angle Group		P
	Mean	SD	Mean	SD	Mean	SD	
OPT/NSL	102.5	7.3	99.4	7.1	103.6	8.4	>0.1
CVT/NSL	105.3	5.4	102.4	7.6	103.9	6.3	>0.1
CVT/OPT	1.4	0.9	1.1	0.5	2.1	0.3	>0.1

## Results

While linear measurements are shown in Table 3 and Table 4, none of the width measurements of the naso-, palato- and glossopharyngeal airways were significantly different (P>0.05). With an increase of vertical facial height, both Ln and Lp were significantly decreased (P<0.05), but there were no significant differences in Lg. Comparing height measurements among the three subgroups with ANOVA and SNK-q test, Hg was significantly less in the high angle group than in low angle or normal angle group (P = 0.022, P = 0.017), but there was no significant difference between low angle and normal angle groups (P>0.05). Although Hn and Hp were less in the high angle group than in either low angle or normal angle groups, this difference was not significant (P>0.05). Altogether, the high angle Class II subjects presented significantly smaller upper airway linear measurements than low angle and normal angle Class II subjects.

**Table 3:** Linear comparison of pharyngeal airway among different vertical facial types (X¯ ± S).

	Subgroups	?X ± S	F	P
	L	35.52±1.85		
Hn (mm)	N	41.93±2.87	1.532	P = 0.073
	H	36.81±1.83		
	L	50.22±1.67		
Hp (mm)	N	45.87±3.24	1.653	P = 0.062
	H	42.59±2.83		
	L	43.55±2.62		
Hg (mm)	N	48.94±2.73	3.217	P = 0.047*
	H	33.13±1.46		
	L	24.16±2.11		
Ln (mm)	N	23.91±2.15	3.452	P = 0.042*
	H	19.88±2.53		
	L	15.04±1.43		
Lp(mm)	N	9.62±2.17	3.57	P = 0.038*
	H	6.72±1.62		
	L	12.14±1.57		
Lg (mm)	N	13.55±3.06	1.385	P = 0.125
	H	10.07±1.39		
	L	28.57±2.82		
Wn (mm)	N	28.47±2.67	1.025	P = 0.386
	H	28.33±3.57		
	L	26.65±4.02		
Wp (mm)	N	23.72±2.65	1.187	P = 0.259
	H	23.28±3.88		
	L	24.77±2.85		
Wg (mm)	N	24.68±3.40	1.503	P = 0.226
	H	22.62±1.38		

L indicates low angle group, N indicates normal angle group, H indicates high angle group. * indicates significant at the 0.05 probability level; NS indicates not significant at the 0.05 probability level.

**Table 4:** Multiple comparison of linear measurements in different vertical facial types.

	Subgroups	Mean	P
	L-H	10.59	P = 0.022*
Hg (mm)	N-H	15.93	P = 0.017*
	L-N	−5.4	P = 0.348
	L-H	4.28	P = 0.035*
Ln (mm)	N-H	4.03	P = 0.035*
	L-N	0.15	P = 0.192
	L-H	8.32	P = 0.008**
Lp (mm)	N-H	2.9	P = 0.086
	L-N	5.42	P = 0.029*

L indicates low angle group, N indicates normal angle group, H indicates high angle group. * indicates significant at the 0.05 probability level; **indicates significant at the 0.01 probability level.

Among volume measurements compared in Table 5, only a difference in Vg among the three subgroups was statistically significant, being less in the high angle group than in low angle or normal angle groups (P<0.05).

**Table 5:** Volume comparison of pharyngeal airway in different vertical facial types (X¯ ± S).

	Subgroups	?X ± S	F	P
	L	6423.07±1328.93		
Vn (mm^3^)	N	5273.98±1172.46	1.274	P = 0.132
	H	5144.67±1033.08		
	L	8372.78±1596.30		
Vp (mm^3^)	N	7664.74±1024.92	1.202	P = 0.204
	H	7323.18±1606.35		
	L	6423.6±1008.75		
Vg (mm3)	N	5997.05±1674.90	3.689	P = 0.037*
	H	4412.96±972.94		
	L	20954.21±3815.02		
Vt (mm^3^)	N	19430.14±1908.15	1.137	P = 0.283
	H	18304.7±3501.51		

L indicates low angle group, N indicates normal angle group, H indicates high angle group. * indicates significant at the 0.05 probability level.

Comparison of cross sectional area measurements in Table 6 showed that CSAn in the high angle group was significantly less than that in low angle or normal angle groups. CSAp was much smaller than CSAg and CSAn, indicating that the inferior border of the palatopharyngeal airway was the most constricted area of the upper airway.

**Table 6:** Comparison of cross sectional area measurements of pharyngeal airway in different vertical facial types (X¯ ± S).

	Subgroups	?X ± S	F	P
	L	729.69±139.02		
CSAn(mm^2^)	N	706.44±94.96	3.715	P = 0.032*
	H	522.64±68.88		
	L	241.65±28.26		
CSAp (mm^2^)	N	271.87±68.25	2.074	P = 0.058
	H	272.89±37.98		
	L	300.21±27.79		
CSAg (mm^2^)	N	358.99±106.52	1.602	P = 0.064
	H	299.62±71.92		

L indicates low angle group, N indicates normal angle group, H indicates high angle group. * indicates significant at the 0.05 probability level.

Finally, we investigated the association between upper airway dimensions and vertical growth patterns (Table 7). It was shown that palato- and glossopharyngeal dimension parameters such as Vp, Vg, Hp, Hg, CSAp and CSAg were significantly positively correlated with ODI, whereas palatopharyngeal dimension parameters such as Vp, Hp and CSAp were significantly negatively correlated with FMA and GoGn-SN angles.

**Table 7:** The association between upper airway dimensions and vertical growth patterns.

	ODI	FMA	GOGN-SN
Vn	0.041	−0.120*	−0.045
Vp	0.451**	−0.252*	−0.264*
Vg	0.175*	−0.09	−0.172*
Vt	0.069	0.05	−0.026
Hn	0.158	−0.121*	−0.143*
Hp	0.205*	−0.250*	−0.072
Hg	0.398*	−0.016	−0.096
CSAn	0.140*	−0.102*	−0.034
CSAp	0.634**	−0.431**	−0.264*
CSAg	0.371*	−0.043	−0.03
Ln	0.120*	−0.017	−0.216 *
Lp	0.047	−0.062	−0.084
Lg	0.041	−0.007	−0.153*
Wn	0.165*	−0.029	−0.126*
Wp	0.05	−0.056	−0.038
Wg	0.067	−0.014	−0.012

* indicates significant at the 0.05 probability level; **indicates significant at the 0.01 probability level.

## Discussion

The relationship between pharyngeal dimensions and the dentofacial pattern in OSA patients has long been of interest to orthodontists. During the past few years, numerous studies have reported on the relevance of pharyngeal dimensions to various sagittal and vertical facial growth patterns at varying degrees [3], [6], [24]. Hyperdivergent patients with certain skeletal features, such as retrusive mandible and vertical maxillary excess, may have narrower anterioposterior airway dimensions [7]. Thus, knowledge of pharyngeal dimensions amongst the various sagittal and vertical facial types is of great importance for orthodontist, especially during orthodontic diagnosis and treatment planning. Furthermore, orthodontic correction of dentofacial abnormalities in adult skeletal class II patients with vertical growth patterns might improve the patient’s life by not only enhancing dentofacial esthetics and function, but also preventing and managing OSA and snoring.

Evaluation of the airway is an essential diagnostic step for OSA patients. In these patients, airway assessment has been conducted primarily on two-dimensional lateral cephalograms by identifying specific landmarks and performing linear measurements in the pharyngeal region [25]. Lateral cephalograms suffer from severe limitations with inherent errors such as 2-dimensional (2D) representation of a distorted 3-dimensional (3D) structure, differences in magnifications, superimposition of bilateral craniofacial structures, and low reproducibility as a result of difficulties in landmark identification [12]. Another important drawback of lateral cephalograms is the lack of information about cross-sectional area and volume. Cone-beam computed tomography (CBCT) provides 3D-reconstructed images from multiple sequential planar projection images, making cross-sectional and volumetric investigations of the upper airway possible. Aboudara et al. [18] demonstrated that CBCT is a simple and effective method to accurately analyze upper airway. They compared the volumetric measurements from CBCT with known physical airway phantoms and found that the errors ranged from 0 to 5%. They also reported a moderately high correlation (r = 0.75) between the sagittal area and volume when correlating lateral cephalograms measurements with CBCT data. The study from Ahmed Ghoneima et al [26] showed that 3D CBCT digital measurements of airway volume and of the most constricted area of the airway are reliable and accurate. Thus, CBCT provides a superior imaging modality in dentalmaxillofacial diagnosis by offering improved conditions for volumetric analysis and accurate visualization of the airway.

It is reported that the size of the nasopharynx correlates closely with skeletal growth and age [27], [28]. Sheng et al. reported that developmental changes occur in the pharyngeal airway from childhood to young adulthood [29]. In this study, all of our patients had no growth potential with a mean age of 26.3 years (range 20–35). Some studies [30], [31] also revealed differences in the pharyngeal airway dimensions of men and women, while others [32]–[35] found no correlation between gender and nasal airway size. Due to inconsistencies in the conclusion of these studies, we aimed to enhance knowledge in the field by controlling for known factors. Sample size was expanded and the distribution of sex and age was controlled, with no significant differences among the three subgroups. The influence of tonsil and adenoid hypertrophy was also limited. Therefore, the interference of interrelated and confounding variables was minimized in this study.

Associations of Class II malocclusions and vertical growth pattern with obstruction of the upper airway and mouth breathing have been reported. Ceylan and Oktay [32] found that changes in the ANB angle may affect nasopharyngeal airway size, and that the oropharyngeal space was reduced in subjects with an increased ANB angle. Similarly, Kerr [36] reported that Class II malocclusion subjects showed narrow nasopharyngeal airway space compared to Class I and normal occlusion subjects. In spite of the above evidence, few studies have addressed pharyngeal airway dimensions in untreated skeletal Class II subjects according to different facial patterns. In fact, several other studies suggest that the vertical dimension may have a stronger effect on airway space than a sagittal jaw relationship. The recent study from Ulas Oz [37]showed that airway space measurements in low- and neutral-angle Class II subjects did not differ from those of the skeletal Class I control group, while the high-angle group’s upper airway space was significantly smaller. De Freitas et al. [38] also found that vertical growth patterns, but not malocclusion type, influenced upper airway dimensions. Ucar and Uysal [39] reported a significant difference between low angle and high angle Class I groups at the level of the nasopharyngeal airway space. The nasopharyngeal airway space decreased from low-angle to normal- to high-angle cases, and they highlighted the effect of the vertical pattern on upper airway space. In terms of our present study, we found that the vertical skeletal pattern had a significant effect on the pharyngeal airway dimensions of untreated skeletal Class II adults.

We showed a significant tendency for reduced pharyngeal airway measurements in high angle subjects. The vertical skeletal pattern did not affect the linear transverse width of the pharyngeal dimension with no significant differences among three subgroups, whereas the linear sagittal length of naso- and palatopharynx (Ln and Lp) was decreased with an increase of the GoGn-SN angle (P<0.05). Accordingly, CSAn in the high angle group was significantly less than that in normal angle or low angle groups, as were Hg and Vg. Vn, Vp and Vt were also less in the high angle group than in low angle or normal angle groups, though the difference was not significant (P>0.05). These data are in line with the findings of a previous study from de Freitas et al. [38] which found that subjects with Class II malocclusions and vertical growth patterns have significantly narrower pharyngeal dimension than those with Class II malocclusions and horizontal growth patterns. Further correlation analysis indicated an association of pharyngeal airway measurements with different vertical growth patterns. Specifically, palato- and glossopharyngeal dimensions were negatively correlated with facial height and the mandibular plane angle in adult skeletal Class II malocclusion subjects. This was a bit inconsistent with the findings of previous studies of Ucar and Uysal [39] who showed that vertical growth patterns have significant correlations with the upper portion of pharyngeal airways. Differences in methods of airway analysis or selection of subjects might contribute to these inconsistencies.

The current study has limitations. Subjects included were Han Chinese adults, who may have morphological features different from other ethnic groups. Measurements were made in an upright position. In reality, the bias brought by a mechanically weak structure such as the pharynx during sleep and even more in sleep apnea should be taken into account.

## Conclusions

The findings of this study on a population of adults showed a smaller airway space in skeletal high angle Class II subjects, thereby, confirming an association between pharyngeal airway space and a vertical skeletal pattern. Moreover, the present study partially demonstrated that vertical growth patterns might predispose a person to pharyngeal narrowing, which in turn might predispose the person to upper airway obstruction. These findings might aid in the orthodontic management of Chinese subjects with obstructive sleep apnea.
